# Physical Connectivity as Enabler of Unexpected Encounters With Information in Campus Development: A Case Study of South China University of Technology

**DOI:** 10.3389/fpsyg.2021.635012

**Published:** 2021-11-02

**Authors:** Yubo Liu, Mian Ji, Qiaoming Deng, Kai Hu

**Affiliations:** ^1^School of Architecture, South China University of Technology, Guangzhou, China; ^2^State Key Laboratory of Subtropical Building Science, South China University of Technology, Guangzhou, China

**Keywords:** interdisciplinary innovation, space syntax, university campuses, physical connectivity, unexpected encounters with information

## Abstract

This paper is an attempt to advance research on spatial potential for interdisciplinary innovation of university campuses by proposing a spatial quantitative method. The aim is to develop the campus to adapt to the new pedagogical structure of encouraging interdisciplinary innovation in the era of knowledge society. For this purpose, literature from management, psychology, and architecture are reviewed to provide insight into the relationship between innovation and physical environment. The existing research mainly focused on the characteristics of physical environment that supported individual innovative thinking or innovative interaction between people in building scale, which is relatively limited in this study for the campus scale since people are less likely to exchange academic information with strangers because of a lack of knowledge about their professional background. In this context, this research enriches the understanding of spatial potential for innovation by proposing a more effective way of increasing unexpected encounters with information, which are probably occurred while people passing by laboratories, seminars, or exhibitions of other disciplines. In this process, the unexpected encounters with information act as the medium or promotion factor for face-to-face interaction. This kind of innovative potential requires fewer conditions like acquaintance or face-to-face interaction but depends more on the space organization. Physical connectivity acts as enabler and the effects vary. This article reports on a preliminary study of how Space Syntax as a quantitative approach is applied to evaluate the effects in the case of South China University of Technology. The proposed method aims to sustain a sustainable transition toward a more adaptable relation between people and the campus environment. However, to improve understanding of spatial effects on innovation, more empirical studies must be carried out.

## Introduction

In the era of knowledge society, universities are expected to evolve from performing conventional research and education functions to adapting to the new pedagogical structure of encouraging interdisciplinary innovation. Since there is a near-universal agreement within the social and architectural literature on the importance that physical environment plays in the innovation process (Allen, 1977; Hillier and Penn, 1991; Penn and Hillier, 1992; Van den Bulte and Moenaert, 1998; Clements-Croome, 2006; Wineman et al., 2009; Stryker et al., 2011; Oksanen and Ståhle, 2013), restructuring the existing campus to achieve the goal of promoting interdisciplinary innovation is a sustainable transition toward a more adaptable relation between people and the campus environment. Existing research on the correlation of organizational innovation and physical environment concentrates on management, psychology, architecture, and so on. Research in psychology and management has mainly focused on how the physical environment supports individual innovative thinking and innovative interaction between people in building scale (Allen, 1977; Amabile, 1996; Allen and Henn, 2007; Oksanen and Ståhle, 2013). As to architecture, studies are based on the findings that interaction can trigger innovation and further explore how spatial organization can promote interaction (Hillier and Penn, 1991, 1992; Wineman et al., 2009; Kabo et al., 2014; Sailer and Thomas, 2019). Space syntax, as a quantitative approach to investigate relationships between spatial layout and social phenomena, is the main method for innovation studies in architecture. Research using space syntax theory has shown how movement patterns are powerfully shaped by spatial layout and how buildings can create more interactive organizational cultures.

In this paper, we are concerned with university campuses and a global perspective on innovation promotion. Since existing studies focused on building scale, the previous approach of promoting face-to-face interaction is relatively limited in this study for the campus scale. Due to the fact that people are less likely to interact with strangers that they meet in the campus, academic information exchange hardly occurs. In this context, this research enriches the understanding of spatial potential for innovation by proposing a more effective way of increasing unexpected encounters with information from other disciplines, which is probably occurred while people passing by laboratories, seminars, or exhibitions of other disciplines. Related research in human information behavior argued that information horizons and information resources are factors that influencing innovation processes (Sonnenwald, 1999; Toker and Gray, 2008). The proposed way of promoting innovation by the unexpected encounters with information is to increase information resources. Encounter with information during one’s movement is passive input of the latest information from different disciplines, which are more likely to trigger interdisciplinary innovation. In this process, the unexpected encounters with information act as the medium or promotion factor for face-to-face interaction. Thus, promoting the unexpected encounters with information complements the existing research that focused on face-to-face interaction, especially in the context of university campuses.

This research is an attempt to explore a spatial quantitative method for evaluating the innovative potential brought by the campus development. This development aims at breaking down the knowledge boundaries of different disciplines and increasing the through-movement to pass by space with information of different disciplines. Inspired by MIT’s “physical connectivity” principle, the goal of creating unexpected encounters with information can be achieved by the spatial strategy of connecting isolated research buildings and integrating them into the network of the whole campus. The key of the method is to evaluate the innovation potential by calculating the effective large-scale through-movement. This article reports on a preliminary study of how Space Syntax as a quantitative approach is applied to the innovation-driven renovation in the case of South China University of Technology. Data collection included the syntactical properties calculated by Depthmap (Turner, 2006) and the practical data by gate counts (Al-Sayed, 2014) from cameras. Different schemes are compared quantitatively and the effects of physical connectivity are discussed. The results of the study approve the adaption of Space Syntax and provide effective spatial strategies for the campus development. Overall, the greatest contributions of this research are to enrich the understanding of the spatial potential for interdisciplinary innovation in campus context and provide a quantitative method for evaluating the innovative potential brought by the campus development.

### Existing Research on the Effects of Physical Environment on Innovation

The effects of physical environment on organizational innovation are explored in many different disciplines such as management, psychology, and architecture. As early as in the late 1970s, Allen had proved the effect of architectural layout on information dissemination through a decade of empirical study into communication behavior among technologists. Allen revealed the exponential drop in frequency of communication between engineers as the distance between them increases, which was graphically presented as “Allen Curve” (Allen, 1977). This discovery bridges the gap between physical environment and work performance by the effect of proximity on communication frequency. Later in 2007, Allen and Henn further explored how organizational structure and physical environment affect communication among people for inspiration that was central to the innovation process (Allen and Henn, 2007). The study illustrated that inter-group communication was more likely to bring innovative problem solving and proximity could be a factor that triggers collaboration. Based on Allen’s pioneering work that proved the importance of communication networks in the Research and Development (R&D) environment for successful innovation, the attention was then turned to the role of communication, cooperation, and integration of R&D with marketing (Griffin and Hauser, 1996). Souder and Moenaert (1992) argued that effective innovation requires various types of knowledge to be mobilized and integrated. Through empirical research, Van den Bulte and Moenaert (1998) demonstrated that communication among R&D teams was enhanced after co-locating these teams. However, the increased physical distance did not affect the frequency of communication between R&D and marketing department. They concluded that the effect of co-location may depend on the content, medium, and strength of the communication flows. Except for physical proximity, the visibility of the work environment and the formal and informal space available for meetings and collaboration are proved to be related to face-to-face communication by a field study conducted at two R&D sites in a large Midwestern United States pharmaceutical company (Stryker et al., 2011). Since new ideas for innovation need to be disseminated rapidly, electronically mediated interactions are growing to replace traditional face-to-face communications. There existed studies comparing the benefits of computer-mediated communication (CMC) and co-location of R&D staff, as well as the mutual interaction between them. Through empirical data collected from 402 high-technology firms in the United States and Netherlands, the results support the effects of the two communication channels on knowledge dissemination, as well as the mutual strengthening role. The research concluded that effective knowledge dissemination required a balance between co-location and information technologies (Song et al., 2007). The space layout typology for collaboration in workplace was further research and the distractions from others’ interactive behavior were considered as well (Hua et al., 2011). Generally, research in the area of management mainly focused on the supporting effect of physical environment on interactive innovation between people. A large number of empirical studies have proved the positive effect of physical environment on innovation through promoting face-to-face communication (Allen, 1977; Van den Bulte and Moenaert, 1998; Hua et al., 2011). However, not all communication can lead to innovation. Innovative interaction requires various knowledge exchange. According to the existing research, the factors of physical environment for supporting innovative interaction include physical distance, visibility, space layout, space type, etc. These factors affect the opportunities of interaction like communication or collaboration thus affects the input of new ideas for innovation.

In the area of psychology, researchers focused more on how physical environment supports individual innovative thinking. The quality of physical environment greatly affects people’s working experience and thus being the most significant factor for innovation. Oksanen and Ståhle (2013) state that physical environment can foster innovation by improving the well-being and happiness of people. “Attractiveness” is one of the five attributes of innovative space presented in her research. She explained attractive space as comforting which affected people’s job performance and satisfaction. Temperatures and noise are revealed to be environmental factors that affect innovative thinking (Clements-Croome, 2006). An attractive physical environment for work is perceived as inspirational and motivational and can trigger innovation (Haner, 2005). Consistent with these findings, Csikszentmihalyi’s study on creativity from the perspective of psychology states that ‘*prepared minds in beautiful settings are more likely to find new connections among ideas and new perspectives on issues dealing with*’ (Csikszentmihalyi, 1997, p. 136). He also discovered that an environment where freedom, security, and control are experienced was beneficial for innovative thinking. Despite comforting environment, stimulating environment can enhance creativity as well. Amabile (1996, p. 249) stated that “physical environments that are engineered to be cognitively and perceptually stimulating can enhance creativity.” Apart from the quality of physical environment, the places and the context affect innovative thinking as well. According to the research of Haynes and Martens (2011) by interviews with creative professionals, places for innovative thinking are diverse. Most people have innovative thinking on moments of relaxation, such as on the way home, while running, under the shower, etc. A similar viewpoint was proposed by Csikszentmihalyi (1997) as well. He stated that “*Just sitting and watching seem fine, but taking a walk is even better*” (1997, p. 137). In general, physical environment can support innovative thinking by providing comforting or stimulating experience. And, many kinds of places are possible to foster innovation, especially on moments of relaxation.

As a specialized discipline studying physical environment, architecture research on the effect of physical environment on innovation is based on the research results of other disciplines, aiming in exploring how to achieve the spatial potential for innovation by design. Since a comforting and attractive environment is the common goal in architectural design, organizing the spatial layout to induce movement that can trigger innovative interaction is the main issue in architecture research. For instance, “generative building” is conceived as being able to increase innovation and creativity since it allows and encourages plurality, contradictions, and dissensus through its spatial organization (Kornberger and Clegg, 2003). Physical proximity is measured with metric distance, topological distance, or geometric distance. Not only the location of staff but also the daily path between different destinations is measured to analyze the degree of overlap (Kabo et al., 2014). Methods applied in these researches include Space Syntax, ArcGIS, flow simulation, and so on, among which Space Syntax is the main force. The purpose of this paper is to explore the spatial possibility for interdisciplinary innovation in the context of university campuses and apply space syntax to construct a spatial quantitative method for innovation-driven renovation.

### Quantitative Research on Organizational Innovation by Space Syntax

Space syntax is an approach built on quantitative analysis and geospatial computer technology to investigate relationships between spatial layout and social phenomena. It is originally proposed by Hillier and Hanson in the 1970s. The approach has developed a set of theories and methods for the analysis of spatial configurations of all kinds and at all scales. Research using the space syntax approach has shown how movement patterns are powerfully shaped by spatial layout and how buildings can create more interactive organizational cultures. Thus, innovation in organizational context is an area to which space syntax theory has contributed.

In “visible colleges,” Hillier and Penn (1991) argued that randomness played a crucial role in the advance of science. They proposed that buildings as organizers of space could act either a conservative or a generative mode. Space with conservative mode leads to the reproduction of existing knowledge, while space with generative mode leads to production of new knowledge. A generative building is depending on relating its spatial structure to randomness. By maximizing randomness of encounters through spatial proximity and movement, new relational patterns like new ideas or new relationships are more likely to emerge. Citing the research of Allen (1977) on the relationship between innovation and communication, as well as Granovetter’s work on social networks in broader community, the paper pointed to the need for a more global view of networks. It is proposed that good urban networks are not self-contained groups but distributions of probabilities within a larger, continuous system (Hillier and Penn, 1991, p. 38). In general, this paper demonstrates how the patterns of space work in the generation of innovation as a social function of buildings, which accounts for the application of space syntax.

In the later paper of Penn and Hillier (1992) “the social potential of buildings,” they focus more directly on the innovative milieu in scientific research laboratories. Based on the findings of Allen (1977) linking innovation to inter-group communications, they bridged the gap between innovative potential of a building and its configuration, which is instrumental in the process of random encounters. Integration was used as a measure of spatial layout to quantify building plans. Through 24 building floors’ studies by means of spatial analysis, observation and questionnaire survey, the paper proved the correlation between spatial integration and the level of useful work-related inter-group communication. The paper further emphasized the significance of movement and recruitment by citing Backhouse and Drew (1992). Hence, they put forward the proposition that innovation requires large-scale movement structure to generate probabilistic interaction between people in different fields and thus break down the boundaries of scientific knowledge.

Similar research on space of innovation has been done in work environment (Penn et al., 1999) as well. A case study of an energy utility was carried out both before and after the company moved to new premises, where spatial analysis assisted the design process. The study found that spatial configuration had an impact on the innovative potential in office-based organizations by affecting the frequency of contact, which was the basis of useful work-related communications.

Despite research focusing on the spatial dimensions involved in the innovation process, there are studies taking social networks into consideration as well. In “spatial and social networks in organizational innovation,” Wineman et al. (2009) examined the effects of spatial layout on social network structure and the support of innovation in a professional school at the University of Michigan, which allocating office space across departments to promote cross-disciplinary collaboration. In this context, the study used co-authored academic publications as the indicator of successful collaboration that reflected the effect of the innovative space. The social network was inferred from the patterns of co-authorship. The study concluded that social (departmental affiliation) and spatial (step-depth) variables do have a significant effect on co-authorship (Wineman et al., 2009, p. 437), and integration could be a spatial measure of innovative potential. Wineman’s study enriched the understanding of innovative potential of space in social dimension by proposing the collaborative interface generated by spatial layout. Collaboration as a social pattern can be directly related to the innovative output by co-authored works, which is more objective than the useful communication by questionnaire surveys in previous research.

In the recent study of Sailer and Thomas (2019), an organization’s innovative potential was quantified according to the “correspondence or non-correspondence” theory, which is an essential description of social-spatial relations by Hillier and Hanson (1984). “Correspondence” describes the high degrees of overlap between spatial and social closeness. This means similar people occupy proximate spaces due to the spatial and social boundaries. In contrast, “non-correspondence” describes an open system that can generate randomness (Hillier and Penn, 1991) and bring different people together across scales. Hence, the paper proposed a way to calculate the degree of correspondence or non-correspondence for the purpose of judging innovative potential. The calculation methodology analyzed the spatial network in depth. Instead of measuring the distance of work locations to establish proximity, this paper defined spatial closeness according to the visible areas of each worker’s daily routine. Those that could be seen in one’s daily routine were potentially encounters and therefore calculated as spatially close. The method was applied to study two work organizations and the results confirmed that non-correspondence system performed better in the spatial promotion of innovation. This means that passing by areas of other fields in daily path makes sense in the process of innovation.

Numerous analytic studies of the structure and functioning of space suggest that the spatial configuration as a property of space on global scale is critical, whether to the structuring of co-presence through movement, or the development of social networks. Space as a physical arrangement acquires its social logic through the encounter probabilities in terms of the frequency and the type of encounters. As innovative problem solving requires importing new information from other fields, large-scale movement that is more likely to pass by work locations of other fields can break down the spatially enforced boundaries between people in different fields. This is the general logic of research on innovation by space syntax.

### The Inspiration of “Physical Connectivity” by MIT

Massachusetts Institute of Technology (MIT) was founded in 1861 and is widely known for its innovation and academic strength. The MIT community aims in making a better world through education, research, and innovation. Its pursuit of innovation is reflected in its campus development as well. Before moving to Cambridge, the MIT campus consisted of dispersed faculties in different city blocks due to the hectic expansion, which was time-consuming and inconvenient for students and faculty. The dispersal of campus layout resulted in the sense of diffusion. Demands for a cohesive collegiate atmosphere become imperative for its preeminent innovative institutional identity. Therefore, the design of the new campus in Cambridge situated the Institute inside a single, massive structure closely resembling the arrangement of the modern factories which pursue efficiency (Jarzombek, 2017; Deng et al., 2019). The emphasis of “physical connectivity” between buildings of different faculty was born. The unified central structure has become the most significant design innovation on campus, which created the inspiring space in the MIT campus: the infinite corridor. It is MIT’s spinal cord that connects most buildings of different disciplines at MIT, including various departments, classrooms, and labs. Since it is the most direct route to disparate parts of the school, it is unsurprisingly the busiest within the campus. It enables conversation and interchange among students, faculty, and staff, which is important for the cross-fertilization of interdisciplinary studies. Besides, the role of the infinite corridor in promoting interdisciplinary innovation was enhanced by renovation projects that created public-facing laboratories, such as the Nano Lab, the Under Graduate Teaching Laboratory, and the Laboratory for Advanced Materials, etc. These Laboratories are equipped with floor-to-ceiling glass walls through which casual passersby could see researchers and students at work. This is how physical connectivity acts as enabler of unexpected encounters with information that promotes innovation. The characteristics of physical connectivity in MIT can be concluded as interconnected, visible and accessible. “Interconnected” requires links between buildings of different disciplines, which promote interdisciplinary communication. “Visible” requires open or transparent segregation along the corridor, which enables the unexpected encounters of information; “Accessible” is the requirement of flow, which will be effective in accordance with students and teachers’ daily routine. Today, the MIT building stands largely unchanged, testifying to the farsighted planning more than 100years ago. Although the Institute’s needs and ideas shifted over the years, the Planning Office of MIT strove to make MIT the great institution “*where an array of ideas are readily available and opinions vigorously articulated*” (Simha, 2001, p. 4). Physical connectivity is considered as the fundamental qualities that nurtured the intellectual and social life of students and faculty in various ways (Simha, 2001). It is also a vital principle in future planning. “MIT 2030” is a living framework that guides the planning activities of MIT campus. “Innovation and collaboration” is one of the themes, which require future planning activities to create the possibility of unexpected connections between people or knowledge in different fields. The proposed physical developments on campus address issues of proximity, potential collaboration, knowledge transfer, and interdisciplinary cross-fertilization. The success of MIT has proved the significance of “physical connectivity” for innovation, which is worthy of reference by other campus planning.

### Interdisciplinary Innovation by Unexpected Encounters With Information in Campus Context

This paper is an attempt to advance research on the spatial potential for innovation in the context of university campuses. The research scope does not cover the whole innovation process but mainly focuses on the introduction of new ideas, which has much to do with space. The adoption and implementation of new ideas remain outside the scope. Literature from management, psychology, and architecture is reviewed. The existing research mainly focused on the characteristics of physical environment that supported individual innovative thinking or innovative interaction between people in building scale. Interaction as a prerequisite of innovation is relatively limited in the context of campus since people are less likely to exchange academic information with strangers because of a lack of knowledge about their professional background. Hence, inspired by the physical connectivity principle in MIT and based on the findings of human information behavior, this paper proposes that unexpected encounters with information from other disciplines can be an effective way to promote interdisciplinary innovation in campus context. It probably occurs while people passing by laboratories, seminars, or exhibitions of other disciplines, where advanced information is visible or displayed on purpose. This kind of innovative potential requires fewer conditions like acquaintance or communication but depends more on the spatial structure and function allocation, in which aspects architecture can intervene. Besides, the unexpected encounters with information may act as the medium or promotion factor for face-to-face interaction. Thus, promoting unexpected encounters with information complements the existing research that focused on face-to-face interaction, especially in the context of university campuses.

Related theories in human information behavior and social psychology account for this proposition. On one hand, it is argued that innovation process is influenced by one’s information horizons, which are formed by the available information resources (Toker and Gray, 2008). Information resources are various, such as social networks, documents, information retrieval tools, and experimentation and observation in the world (Sonnenwald, 1999). The proposed way of promoting unexpected encounters with information from other disciplines is to increase information resources. There are active and passive inputs of information. Except for interacting with people, active information seeking is limited within one’s scope. Encounter with information during one’s movement is passive input of information from different disciplines, which is more likely to bring innovative ideas. The findings of information behavior also indicate that such unexpected encounters with information have more chances to promote innovation in terms of one’s daily routes. This is because the absorption of information follows the principle of minimum effort. And people are more likely to have innovative thinking on moments of relaxation, such as on the way home. On the other hand, the visible stimulation like the display of research results or working status can create an atmosphere of competition, which is a kind of creative context (Amabile, 1996).

Accordingly, the spatial strategy that we adopt in the innovation-driven development of university campuses is to construct physical connectivity between isolated research buildings and integrate the indoor traffic flow into the network of the whole campus. How to maximize the daily movement passing through spaces with information is the key to spatial organization. The innovation-driven development aims at breaking down the knowledge boundaries of different disciplines and increasing the through-movement to pass by the space like labs, exhibitions, or seminars. This spatial strategy of connecting various disciplinary buildings is to improve the environmental heterogeneity, which enables people to gain new perspectives of thinking (Qiao, 2010). This paper attempts to explore a spatial quantitative method for the innovation-driven development of university campuses. Based on the enhancing understanding of spatial potential for interdisciplinary innovation, the application of space syntax is discussed and developed. The key of the method is to evaluate the innovation potential by calculating the effective large-scale movement through spaces with information. Compared to the existing studies, in this context the overlap degree of movement and information is calculated instead of that of different movements. The next chapter will detail how we apply space syntax for this endeavor.

## Preliminary Application Study in South China University of Technology

**Figure 1:**
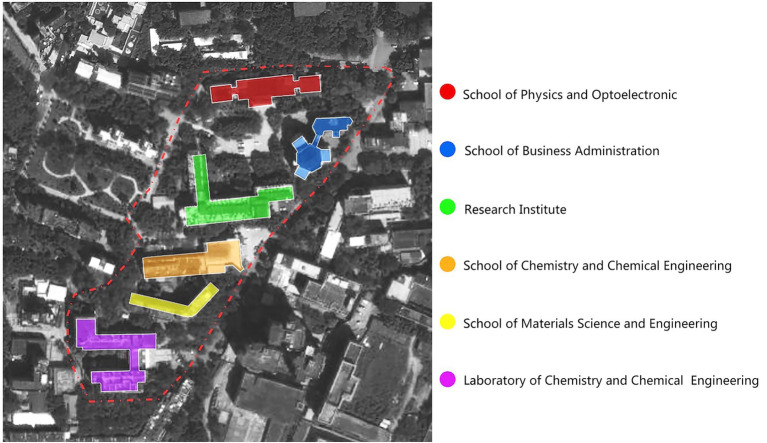
The research district in SCUT campus.

This article reports on a preliminary study of applying space syntax to the innovation-driven development of a research district in the campus of South China University of Technology. There are six isolated buildings of different departments inside the district, which mainly afford offices, classrooms and laboratories for different disciplines (Figure 1). In the previous chapter, we have already reviewed the relevant research by space syntax to highlight its potential for understanding innovation of space. However, most studies focused on one organization within a building. As to campus, it is more like part of the urban network and the social pattern is more complicated. This requires constructing a larger urban network model to estimate and visualize movement performance. Our application study includes two parts. The first part is to construct the network performance model of the whole campus through the correlation analysis of syntactic measures and observation data from cameras. The strongest predictor of the syntactic measures is figured out and lays the foundation for the further analysis of renovation schemes. Other movement attractions like transport and land use are also discussed in the formation of the actual movement performance within the campus. In the second part, according to the aim of integrating the isolated buildings into the network of the campus for promoting innovation by encounters of people and information, we develop different schemes with physical connectivity for the selected research district. We compare the schemes based on quantitative analysis and propose relatively effective spatial strategies for innovation-driven development of the campus.

### Constructing the Network Performance Model of the Campus

**Figure 2:**
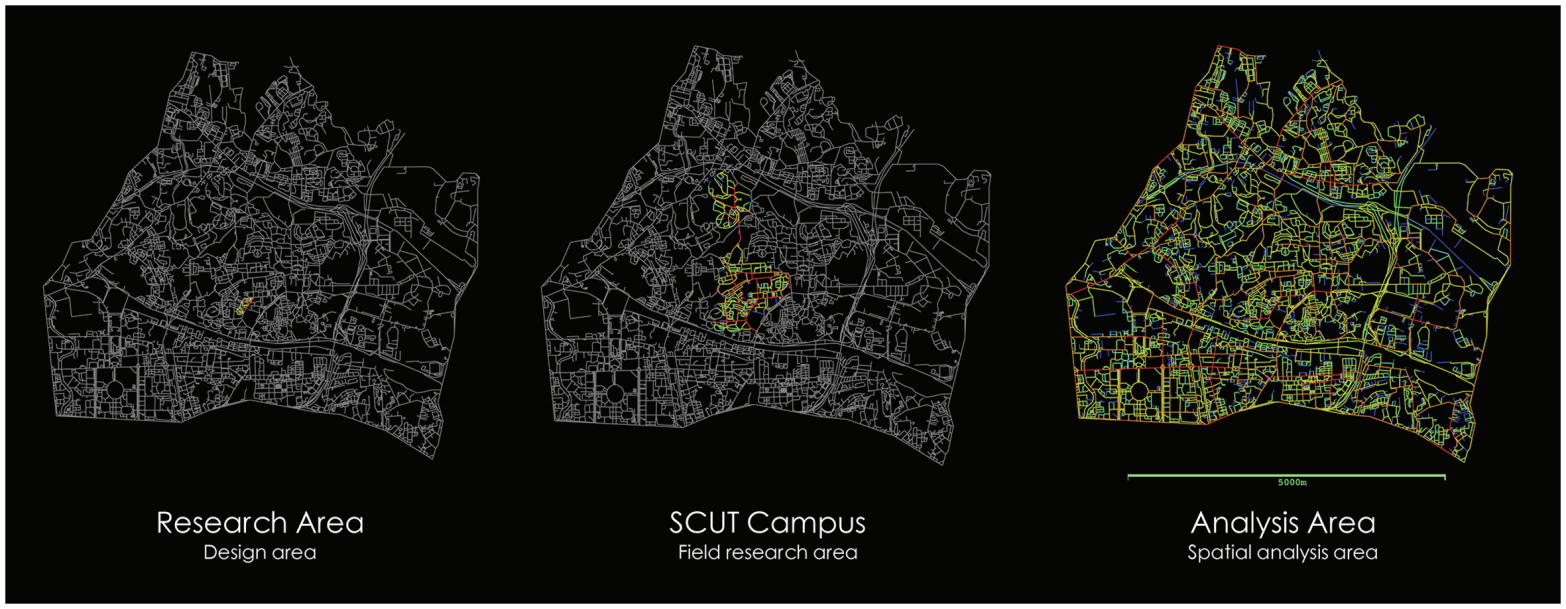
The study area.

**Figure 3:**
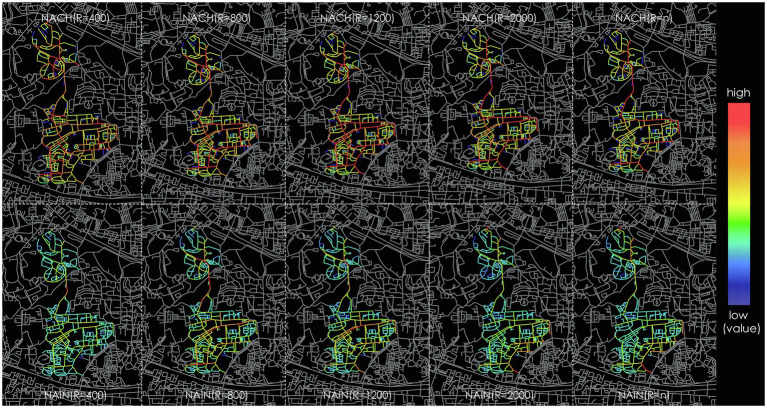
The normalized integration and choice at radius 400m, 800m, 1,200m, 2,000m, and *n*.

The construction of network performance model is particularly relying on angular segment analysis with metric radius, which is a powerful tool for measuring accessibility in street networks and thus predicting social activities (Al-Sayed, 2014). This analysis is on the level of street segments, considering their topological, metric and angular connections. Using this type of segment graph, spatial measures will be calculated to measure accessibility and compare configurational properties of space with observed urban activity. According to the space syntax methodology of requiring a larger area for analysis, we define the boundary of the study area roughly 2,000m from the site (Figure 2) and create the segment map for spatial configuration analysis. The analysis is conducted in Depthmap. Several global and local spatial measures are calculated, including integration and choice at different radius. Both integration and choice are the most powerful syntactic properties in both analysis and movement prediction (Hillier et al., 2012). Integration represents the to-movement potential of a space and choice represents the through-movement potential. In our study, we calculate the integration and choice at radius 400m, 800m, 1,200m, 2,000m, and *n* as global radius (Figure 3). All the measures are normalized to permit comparison of different models. Figure 3 shows the graph results of the analysis. Each segment has its calculated value that can be export for further statistical analysis. “NACH” is short for normalized angular choice. “NAIN” is short for normalized angular integration. The 10 graphs represent the calculated value of “NACH” and “NAIN” at different radius. The colorful segments represent the streets within the campus. Different color represents different degree of the value. The warmer the color the higher of the value. In other words, the red segments are the most accessible streets and predicting the largest flow. The results of the 10 graphs will then be compared with the observed data to define the best predictor.

In order to measure real movement and test spatial predictors, field observation was conducted. Observation techniques are field research methods of space syntax to construct a quantitative description of the movement behavior in the public realm. In this study, we applied gate counts as the method, which was usually directed to observe the density of pedestrian or vehicular movement flows in an urban layout (Al-Sayed, 2014). Our sample consisted of 35 gates being observed for 5min every 2h from 8AM to 5 PM (8:30–8:35, 10:30–10:35, 12:30–12:35, 14:30–14:35, and 16:30–16:35) during both working days and weekends. The observation covered the peak flow period of just before or after class and lunchtime, as well as the trough period of during class. We focused on the non-vehicle movement since both walking and biking were main transportations in campus (Sun, 2019). As most space syntax research, we transformed the movement data by logarithm. Then, we performed correlation and linear regression analysis on the spatial results of the segment analysis above of each radius with the real movement data (Tables 1 and 2). As a result, the normalized choice at radius 1,200m showed the highest correlation and adjusted *R*^2^ value, which indicates the segment angular choice at 1200m as the best predictor that can predict 57.6% of the real movement.

**Table 1:** Results of the correlation analysis of spatial analysis results and real movement data by SPSS.

		Normalized angular choice (NACH; *R*=400)	NACH (*R*=800)	NACH (*R*=1,200)	NACH (*R*=2,000)	NACH (*R*=n)	Normalized angular integration (NAIN; *R*=400)	NAIN (*R*=800)	NAIN (*R*=1,200)	NAIN (*R*=2,000)	NAIN(*R*=*n*)
Log movement average	Pearson Correlation	0.404^*^	0.654^**^	0.767^**^	0.753^**^	0.667^**^	0.398^*^	0.470^**^	0.439^**^	0.236	0.172
	Sig. (2-tailed)	0.016	0.000	0.000	0.000	0.000	0.018	0.004	0.008	0.172	0.324
	N	35	35	35	35	35	35	35	35	35	35

* *Correlation is significant at the 0.05 level (2-tailed)*.

** *Correlation is significant at the 0.01 level (2-tailed)*.

**Table 2:** The linear regression analysis of NACH (R=1,200) and real movement data by SPSS.

Model		Unstandardized coefficients		Standardized coefficients	*t*	Sig.	*R* square	Adjusted R square	*F*
		B	Std. error	Beta					
1	(Constant)	0.877	0.276		3.172	0.003^*^	0.589	0.576	47.264 (0.000^**^)
	NACH(R=1,200)	1.584	0.230	0.767	6.875	0.000^**^			

* *Correlation is significant at the 0.05 level (2-tailed).*

** *Correlation is significant at the 0.01 level (2-tailed).*

*Dependent variable: log movement average*.

*Predictors: (constant), NACH(R=1,200)*.

**Figure 4:**
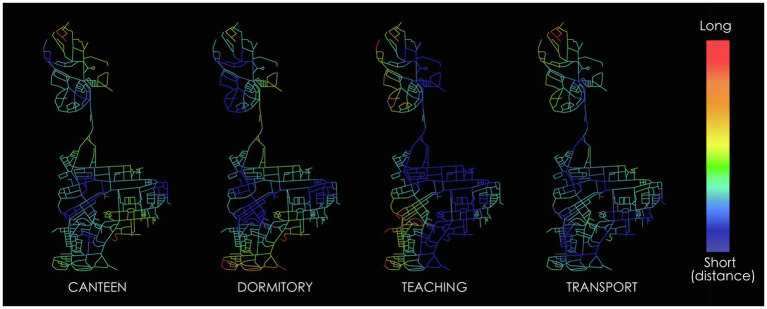
Quantify different attractions by metric step depth analysis.

**Figure 5:**
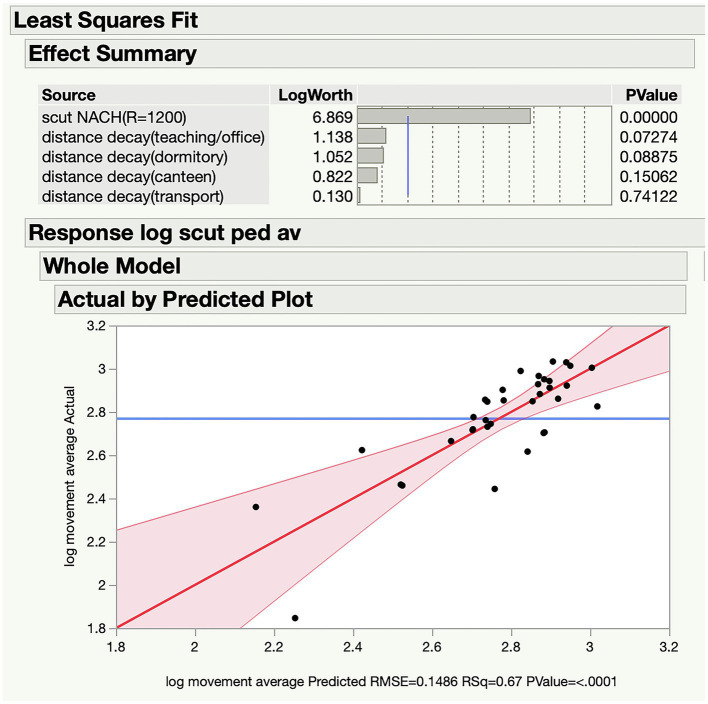
Multiple variable regression analysis in JMP.

The segment analysis quantified the accessibility of each street according to the configuration of the whole network, which reflects the movement distribution to some extent. Besides, transport station and some land use can affect the movement as well. For instance, once there is a bus station or an attractive shop on the street segment, it will attract much flow even in a relatively segregated position. Transport station and some land use act as movement attractor to some extent. Therefore, we further processed the transport and land use attraction measures to narrow the gap between the segment map and the real movement. We conducted the survey of the distribution of bus stops and land use on the campus. The land use attraction we tested in this study included teaching (include labs, offices, classrooms, and seminars), students’ dormitories, and canteens. We used the metric step depth function in Depthmap to quantify the attraction of both transport and land use. Metric step depth analysis measures the walking distances from each origin to all segments in the network. Origins are the bus stops and the land use attraction that we surveyed before. Figure 4 shows the graph results of the metric step depth analysis of different attractors. The color of the segments represents the calculated distance from attractors. The warmer the color the longer the distance. The distance of each segment calculated in Depthmap was transformed into the attractive ability of movement according to the distance decay. It means that the effect of movement attraction on segments is decreased with the increase of distance from the origins. Having prepared the data of transport and land use attraction for each segment, we performed multiple variable regression analysis with the data of normalized choice at radius 1,200m and real movement in JMP (statistical software). The statistical analysis demonstrated that teaching, students’ dormitories, and canteen are the most effective land use attractions. Combined with these three factors, the *R*^2^ value of the regression analysis increases from 0.576 to 0.67, which means the explanatory power of the spatial accessibility is improved (Figure 5). However, due to the limitation of small sample size, the values of *p* of the attractions do not reach the threshold for significance. Even though, there are conclusions that we can draw from this analysis. On one hand, it demonstrates that spatial configuration (represented by normalized choice at radius 1,200m) plays a significant role in the real movement performance of campus, just like most urban network. On the other hand, unlike urban environment where transport and commerce are always effective attractions, the movement in campus is more likely to be affected by the distribution of teaching and living areas.

### Comparison of Different Schemes for Innovation-Driven Development

In this study, we attempt to link research outcomes with design interventions and introduce an evidence-based process into design. The innovation-driven development of campus from the perspective of space syntax aims in improving the innovative potentiality of space through increasing random encounters of people and information in different fields. The design interventions are thus required to connect buildings of multiple disciplines and integrate the indoor circulation into the public transport network of the campus. Alternative solutions are simultaneously tested by quantitative analysis as the evidence-based process to propose effective design interventions.

**Figure 6:**
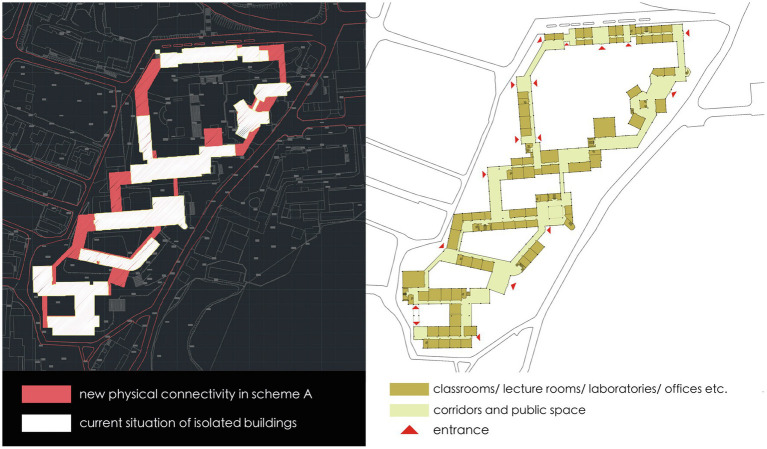
The plan of scheme A which connects isolated buildings on both ends.

**Figure 7:**
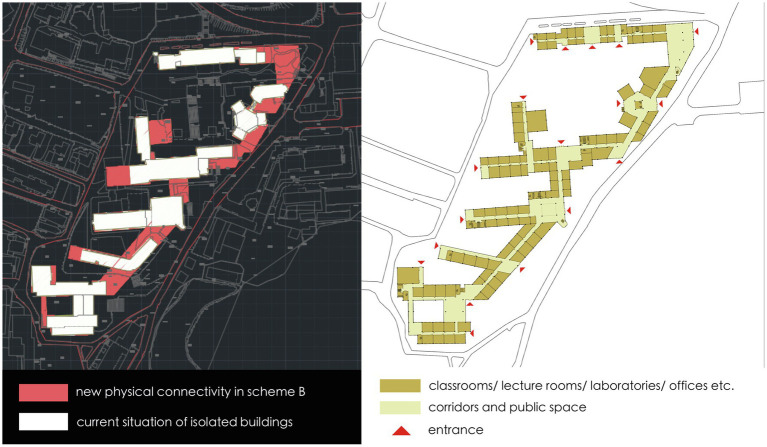
The plan of scheme B which connects isolated buildings on one end.

In Scheme A, the basic concept is to well connect the isolated buildings within the district to form an integrated group, which is achieved by the connection on both ends of each building. The shared entrances are placed in the newly connective part to organize the circulation routes, which not only integrates the group into the public network, but also maximize the overlap of movement to different disciplinary buildings (Figure 6). As to Scheme B, it focuses on global movement performance and the design interventions aim in organizing the indoor circulation routes to enhance the existing network. Therefore, the newly built part concentrates on the east connection of all buildings, which is parallel to one of the busiest and mixed roads in the campus. Such an indoor corridor can efficiently separate pedestrian flow from the existing road and attract through-movement into the research buildings. The entrances are located on the end of each building so that the existing corridors are transformed into direct east–west links across the site and well connected with the existing intersections on the west (Figure 7). With a different starting point, two schemes are generated into different spatial structures. How they perform is further analyzed quantitatively on different scales and effective spatial strategies for innovation-driven renovation are proposed.

**Figure 8:**
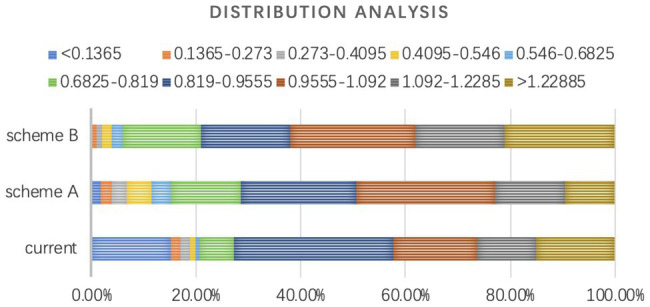
Distribution analysis of calculated results (NACH, *R*=1,200m, segment map).

**Figure 9:**
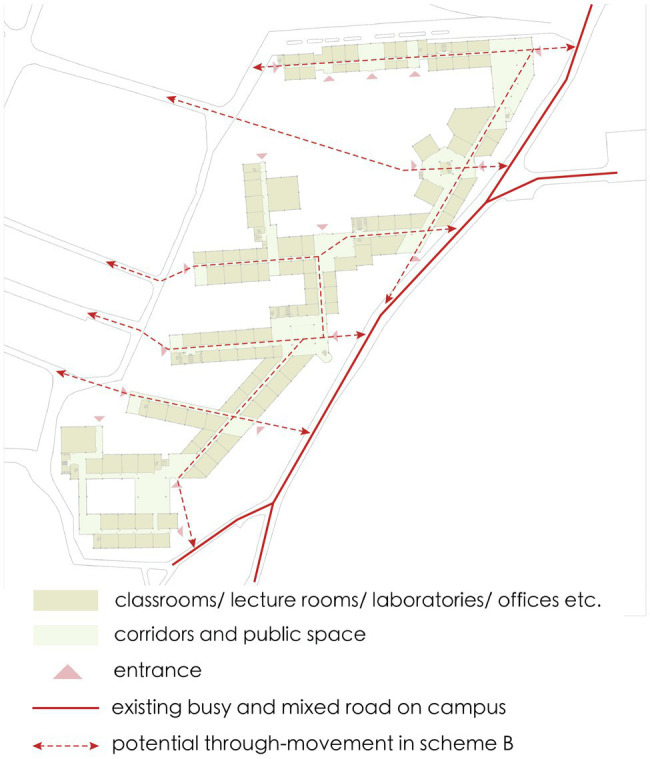
Scheme B organizing the through-movement based on the existing busy road.

On global scale, angular segment analysis is conducted to compare the effect of design interventions on the introduction of through-movement into the research buildings. The analysis is conducted with the model constructed in the previous part and the value of normalized choice at radius 1,200m as the strongest predictor of real movement is used to evaluate the schemes. Table 3 presents the calculated results of the segments within the site for the current situation and two design schemes from Depthmap. The value of normalized choice at radius 1,200m (NACH, *R*=1,200) represent the amount of movement, which indicates the spatial potential for unexpected encounters with information that will probably trigger interdisciplinary innovation. The table shows that both the average value of scheme A and scheme B are increased, indicating that both design interventions succeed in introducing through-movement to the buildings. Compared to the value of the current situation, the average value of scheme A increases by 9.4%, while that of scheme B increases by 20.41%. This means that scheme B performs much better than scheme A in attracting through-movement to the research buildings on global scale, which may generate more random encounters that will trigger innovation. As to the max value and standard deviation, scheme A and scheme B shows a similar trend. The max value of two design schemes are decreased lightly since through-movement is no longer concentrated in two or three routes due to the enhancement of network by design interventions. Thanks to the connection of isolated buildings and the integration of indoor circulation into the public network, both schemes greatly decrease the standard deviation, which is rather high because of so many segregate segments before. The distribution of calculated results is further analyzed (Figure 8; Table 4). Compared to the current situation, scheme B performs better than scheme A in both increasing the percentage of segments with high value and decreasing the percentage of segments with low value. Although scheme A decreases the percentage of segregate segments as scheme B, it does not increase the percentage of segments in the range of relatively high value. To summarize, both the design interventions of scheme A and scheme B have positive effect on the promotion of encounters of people and information by the introduction of through-movement on global scale of the campus. According to the contrastive analysis of calculated results of normalized choice at radius 1,200m, both the average value and frequency distribution reveal that scheme B performs better than scheme A on global scale of the campus, indicating that organizing direct routes across the site to enhance the existing network based on the real movement performance is a relatively effective spatial strategy for innovation-driven development on global scale of the campus. Figure 9 explains how scheme B organizing the physical connectivity to enhance the through- movement network based on the existing busy road on campus. According to the observation data, the road on the west of the site is one of the busiest and mixed roads on the campus. On one hand, scheme B organized a corridor parallel to it that can attract pedestrian from the existing busy and mixed road. On the other hand, entrances are located on the end of each building so that the existing corridors are transformed into direct east–west links across the site and well connected with the existing intersections on the west. The corridors are becoming part of the campus public network that significantly increase the through- movement, which promote unexpected encounters with information that may trigger interdisciplinary -innovation.

**Table 3:** General comparison of the segment analysis (NACH, R=1,200m).

	NACH (*R*=1,200m) of the current situation	NACH (*R*=1,200m) of the scheme A	The increase percentage of scheme A compared to the current situation (%)	NACH (*R*=1,200m) of the scheme B	The increase percentage of scheme B compared to the current situation (%)
Average	0.832521	0.910811	9.40	1.00245	20.41
Max.	1.36536	1.36238	−0.22	1.33625	−2.13
Std. dev.	0.417651	0.272837	−34.67	0.223965	−46.38

**Table 4:** Distribution comparison of the calculated results.

Value of NACH (*R*=1,200m)	Percentage of segments amount of the current situation (%)	Percentage of segments amount of scheme A (%)	Percentage of segments amount of scheme B (%)
<0.1365	15.09	1.90	0.00
0.1365-0.273	1.89	1.90	1.00
0.273-0.4095	1.89	2.86	1.00
0.4095-0.546	0.94	4.76	2.00
0.546-0.6825	0.94	3.81	2.00
0.6825-0.819	6.60	13.33	15.00
0.819-0.9555	30.19	21.90	17.00
0.9555-1.092	16.04	26.67	24.00
1.092-1.2285	11.32	13.33	17.00
>1.22885	15.09	9.52	21.00

**Figure 10:**
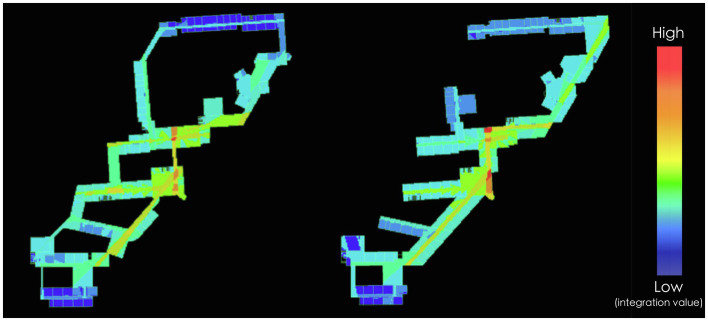
Comparison of visibility graph analysis on local scale (integration, *r*=*n*).

On local scale, the visibility graph analysis is conducted within the group of buildings. Visibility graph analysis is another type of representation of spatial configuration based on visual relationship of space. It helps to understand the visual perception of the built environment and contribute to forecasting how accessible spaces afford movement (Al-Sayed, 2014). Compared to the segment analysis, visibility graph analysis explains a high resolution picture of the spatial configurations of a layout, which is more detailed and suitable for indoor space analysis. In visibility graph, the space is divided into grids, each of which is a unit for analysis. As Penn argued in a study with a sample of 24 building floors (1991), the degree of spatial integration predicted the strength of the network significantly and related to the level of useful work that contributed to inter-group communication. Therefore, integration is used as the syntactic property to evaluate the innovative potential within the building floors at local scale. In general, loop layout with little end space will have higher integration than tree layout. However, the results (Figure 10, Table 5; the warmer the color, the high the integration value/the better accessibility) reveal that although buildings of different disciplines are well connected by loop circulations in scheme A, its performance of integration does not show a distinct advantage over scheme B, in which buildings are connected on one end. Despite the small advantage in average value, the minimum, maximum and standard deviation value of scheme A are worse than that of scheme B. The difference of minimum and maximum value between two schemes is relatively distinct, which means that both the most integrated and segregated spaces in scheme B perform much better than scheme A. This analysis of local scale shows that the number of connections between isolated buildings is admittedly meaningful, but the effect on innovative promotion by increasing through-movement also has much to do with how the connections are organized. According to the results, on local scale scheme B performs better than scheme A for two reasons. On one hand, the physical connectivity across buildings of multiple disciplines is straighter that act as a spinal cord of the whole district, while in scheme A physical connectivity are relatively limited between every two buildings. The heterogeneity of physical connectivity affects the chance of unexpected encounters with information from other disciplines thus affecting interdisciplinary innovation possibility. On the other hand, in scheme B the expansion space concentrates in the main physical connectivity that are much visible and accessible for passersby all over the district, while in scheme A the expansion space allocates to physical connectivity between every two buildings like accessory space of different disciplinary. Accordingly, the characteristics of physical connectivity that affect the to-movement potential include not only the amount (in scheme A isolated buildings are connected on both ends, in scheme B isolated buildings are connected on one end), but also the form and the alongside space organization. Constructing spaces along the connective corridors to enhance the connection leads to better performance than merely connective corridors between different buildings.

**Table 5:** Comparison of the visibility graph analysis on local scale (integration, *R*=*n*).

	The integration (*R*=*n*) value of Scheme A calculated by visibility graph analysis	The integration (*R*=*n*) value of Scheme B calculated by visibility graph analysis
Average	3.00933	2.95318
Minimum	1.59817	1.82412
Maximum	4.99472	5.30263
Standard deviation	0.650529	0.638936

## Discussion and Conclusion

This work advances the research on spatial potential for interdisciplinary innovation and provides a spatial quantitative method for guiding innovation-driven development in the context of university campuses. Previous studies in various areas have proved the effect of physical environment on innovation. However, they mainly focused on how physical environment can support innovative interaction within a building, which is relatively limited in this work due to the low possibility of interaction with strangers on the campus scale. Based on the inspiration of the “physical connectivity” in MIT and the related theories in human information behavior and social psychology, the paper enriches the understanding of spatial potential for interdisciplinary innovation by proposing that unexpected encounters with information can trigger innovative problem-solving as well. In order to explore a spatial quantitative method to apply this proposition to the innovation-driven development of university campuses, this paper reviews and develops the application of Space Syntax. In this work, the key of the method is to evaluate the innovation potential by calculating the effective large-scale movement through spaces with information. Compared to the existing studies, in this context the overlap degree of movement and information is calculated instead of that of different movements.

In the preliminary application study, we have presented evidence for the dominance of spatial configuration in the movement performance within the campus of South China University of Technology, which proves the application of space syntax to the study. We also discussed movement attractions and found that unlike urban environment where transport and commerce were always effective attractions, the movement in campus was more likely to be affected by the distribution of teaching and living areas. Alternative solutions for innovation-driven development were simultaneously tested by quantitative analysis as the evidence-based process to propose effective design interventions. Scheme A and scheme B presented a rich comparison of the design process and its outcome of different innovative potential by spatial configuration. Starting with quite similar briefs, their spatial translations were different. Through the quantitative analysis on global and local scale, the study showed that the design briefs of connecting buildings of different disciplines and integrating the group into the campus public network had the spatial affordances to induce random encounters that would trigger and accommodate innovation, but the effect differed from different interventions. It was demonstrated that organizing direct routes across the site to enhance the existing network based on the real movement performance was more effective than concentrating in the connection of isolated buildings to construct an integrated group within the site. For instance, providing parallel routes to the existing busy and mixed roads around the site can effectively separate pedestrian flow and attract through-movement into the research buildings. Besides, extending existing streets that block by the site and transforming existing indoor corridors into direct link across the site is also effective solutions to enhance existing network as innovation-driven spatial strategies. Moreover, the visibility graph analysis within the group of buildings indicated that connections between isolated buildings contributed to the innovative potential by increasing through-movement in terms of connectivity amount, space form and alongside space organization.

To summarize, this research explores potential opportunities to guide the campus development to create more innovative work processes. In previous studies, innovations are predicted by connecting disconnected individuals and enhancing coordination between connected individuals (Hillier and Penn, 1991; Wineman et al., 2009; Sailer and Thomas, 2019). This paper enhances the possibility of innovation by promoting unexpected encounters with information. Physical connectivity as enabler is discussed in terms of its effect on organizing the global through-movement and transforming the spatial configuration of the local indoor space. This area of exploration has broader impacts to campus administrators, research space designers and architects in producing innovative work environment. Spatial layout is often considered a powerful tool for shaping organizational culture and achievement. And physical connectivity in the campus context can be a useful strategy to adjust the spatial layout to respond to the new pedagogical structure of encouraging interdisciplinary innovation.

Limitations of this application study are mainly derived from the small sample of field research data. Due to the Covid-19 pandemic, we could not obtain more data of the real movement in campus. Moreover, it is crucial to support this study with investigations of the actual performance of the buildings, which might differ according to the daily operation of the building, the management and many other factors. Therefore, further research may continue to study the academic and social life inside the buildings as well as activity patterns, and relate this to the innovative potentiality afforded by configuration.

## Data Availability Statement

The original contributions presented in the study are included in the article/supplementary material; further inquiries can be directed to the corresponding authors.

## Author Contributions

YL and QD contributed to the conception and framework of the study. MJ wrote the first draft of the manuscript. KH was actively involved in the application study. All authors contributed to the article and approved the submitted version.

## Funding

This research is supported by the National Natural Science Foundation of China (nos. 51978268 and 51978269) and the State Key Lab of Subtropical Building Science (no. 2019ZA01).

## Conflict of Interest

The authors declare that the research was conducted in the absence of any commercial or financial relationships that could be construed as a potential conflict of interest.

## Publisher’s Note

All claims expressed in this article are solely those of the authors and do not necessarily represent those of their affiliated organizations, or those of the publisher, the editors and the reviewers. Any product that may be evaluated in this article, or claim that may be made by its manufacturer, is not guaranteed or endorsed by the publisher.

## References

[ref1] Allen T. J. (1977). Managing the Flow of Technology. Cambridge/London: MIT Press.

[ref2] Allen T. Henn G. (2007). The Organization and Architecture of Innovation. New York: Routledge.

[ref3] Al-Sayed K. (2014). Space Syntax Methodology. London: Bartlett School of Architecture, UCL.

[ref4] Amabile T. M. (1996). Creativity in Context: Update to the Social Psychology of Creativity. Boulder: Westview Press.

[ref5] Backhouse A. Drew P. (1992). The design implications of social interaction in a workplace setting. Environ. Plann. B Plann. Des. 19, 573–584. doi: 10.1068/b190573

[ref6] Clements-Croome D. (Ed.) (2006). Creating the Productive Workplace. Oxford, England: Taylor & Francis.

[ref7] Csikszentmihalyi M. (1997). Flow and the Psychology of Discovery and Invention. Harper Perennial, New York, 39.

[ref8] Deng Q. M. Liu Y. B. Simha O. R. (2019). Study no.7 and MIT centennial campus in Cambridge: planning and construction of high efficient campus from perspective of engineer. The Architect 2019, 70–75.

[ref9] Griffin A. Hauser J. R. (1996). Integrating R&D and marketing: a review and analysis of the literature. J. Prod. Innov. Manage. 13, 191–215. doi: 10.1111/1540-5885.1330191

[ref10] Haner U. E. (2005). Spaces for creativity and innovation in two established organizations. Creat. Innov. Manag. 14, 288–298. doi: 10.1111/j.1476-8691.2005.00347.x

[ref11] Haynes B. P. Martens Y. (2011). Creative workplace: instrumental and symbolic support for creativity. Facilities 29, 63–79. doi: 10.1108/02632771111101331

[ref12] Hillier B. Hanson J. (1984). The Social Logic of Space. Cambridge: Cambridge University Press.

[ref13] Hillier B. Penn A. (1991). Visible colleges: structure and randomness in the place of discovery. Sci. Context. 4, 23–49. doi: 10.1017/S0269889700000144

[ref14] Hillier W. R. G. Yang T. Turner A. (2012). Normalising least angle choice in Depthmap- and how it opens up new perspectives on the global and local analysis of city space. Journal of Space Syntax 3, 155–193.

[ref15] Hua Y. Loftness V. Heerwagen J. H. Powell K. M. (2011). Relationship between workplace spatial settings and occupant-perceived support for collaboration. Environ. Behav. 43, 807–826. doi: 10.1177/0013916510364465

[ref16] Jarzombek M. M. (2017). Designing MIT: Bosworth's New Tech Cambridge/London: the MIT Press.

[ref17] Kabo F. W. Cotton-Nessler N. Hwang Y. Levenstein M. C. Owen-Smith J. (2014). Proximity effects on the dynamics and outcomes of scientific collaborations. Res. Policy 43, 1469–1485. doi: 10.1016/j.respol.2014.04.007

[ref18] Kornberger M. Clegg S. (2003). The architecture of complexity. Cult. Organ. 9, 75–91. doi: 10.1080/14759550302804

[ref19] Oksanen K. Ståhle P. (2013). Physical environment as a source for innovation: investigating the attributes of innovative space. J. Knowl. Manag. 17, 815–827. doi: 10.1108/JKM-04-2013-0136

[ref20] Penn A. Desyllas J. Vaughan L. (1999). The space of innovation: interaction and communication in the work environment. Environ. Plann. B Plann. Des. 26, 193–218. doi: 10.1068/b4225

[ref21] Penn A. Hillier B. (1992). “The social potential of buildings, spatial structure and the innovative millieu in scientific research laboratories,” in Corporate Space and Architecture Conference, July 30 - June-3, 1992 (Lyons, Nantes, Paris), 39–43.

[ref22] Qiao H. (2010). Human Behavior. Beijing: Beijing Normal University Publishing Group.

[ref23] Sailer K. Thomas M. (2019). “Correspondence and Non-correspondence. Using office accommodation to calculate an organization's propensity for new ideas.” in *Proceedings of the 12th International Space Syntax Symposium. International Space Syntax Symposium*; July 8–13, 2019 (Beijing).

[ref24] Simha O. R. (2001). MIT Campus Planning, 1960-2000: An Annotated Chronology. Cambridge/London: MIT Press.

[ref25] Song M. Berends H. Van der Bij H. Weggeman M. (2007). The effect of IT and co-location on knowledge dissemination. J. Prod. Innov. Manag. 24, 52–68. doi: 10.1111/j.1540-5885.2006.00232.x

[ref26] Sonnenwald D. H. (1999). “Evolving perspectives of human information behavior: contexts, situations, social networks and information horizons.” in *Exploring the Contexts of Information Behavior: Proceedings of the Second International Conference in Information Needs*; August 13–15, 1998 (London, UK: Taylor Graham).

[ref27] Souder W. E. Moenaert R. K. (1992). An information uncertainty model for integrating marketing and R&D personnel in new product development projects. J. Manag. Stud. 29, 485–512. doi: 10.1111/j.1467-6486.1992.tb00675.x

[ref28] Stryker J. B. Santoro M. D. Farris G. F. (2011). Creating collaboration opportunity: designing the physical workplace to promote high-tech team communication. IEEE Trans. Eng. Manag. 59, 609–620.

[ref29] Sun T. T. (2019). Spatial Distribution of Human Flow in University Campus Based on Space Syntax. Master’s thesis. Guangzhou: South China University of Technology.

[ref30] Toker U. Gray D. O. (2008). Innovation spaces: workspace planning and innovation in US university research centers. Res. Policy 37, 309–329. doi: 10.1016/j.respol.2007.09.006

[ref31] Turner Alasdair. (2006). Ucl Depthmap: Spatial Network Analysis Software. London: University College London, VR Centre of the Built Environment.

[ref32] Van den Bulte C. Moenaert R. K. (1998). The effects of R&D team co-location on communication patterns among R&D, marketing, and manufacturing. Manag. Sci. 44, S1–S18. doi: 10.1287/mnsc.44.11.S1

[ref33] Wineman J. D. Kabo F. W. Davis G. F. (2009). Spatial and social networks in organizational innovation. Environ. Behav. 41, 427–442. doi: 10.1177/0013916508314854

